# Mould deterioration monitoring of Citri Reticulatae Pericarpium using Vis/NIR imaging and an improved Inception ResNet

**DOI:** 10.3389/fpls.2026.1883465

**Published:** 2026-07-22

**Authors:** Chao Ma, Mingkun Zhang, Sen Wang, Zhenzhen Chen, Sudan Chen, Yuxiang Li, Shaowen Jing, Mingtong Du, Jianwei Ma

**Affiliations:** 1 College of Information Engineering, Henan University of Science and Technology, Luoyang, Henan, China; 2 Guangdong HUST Industrial Technology Research Institute, Huazhong University of Science and Technology, Dongguan, Guangdong, China; 3 Artificial Intelligence Department, Yuxin Electronic Technology Group Co., Ltd., Zhengzhou, China; 4 College of Horticulture and Plant Protection, Henan University of Science and Technology, Luoyang, Henan, China; 5 College of Computing and Data Science, Nanyang Technological University, Singapore, Singapore

**Keywords:** Citri Reticulatae Pericarpium, deep learning - artificial intelligence, improved Inception ResNet, mould deterioration, non-destructive detection, Vis/NIR multispectral imaging

## Abstract

**Introduction:**

Mould deterioration is a critical quality risk for Citri Reticulatae Pericarpium (CRP) during storage, reducing its commercial value and potentially compromising the safety of dried food and food–medicine homologous products. Conventional visual inspection is subjective and may fail to identify early deterioration.

**Methods:**

Visible and near-infrared (Vis/NIR) multispectral imaging was combined with deep learning to develop a rapid and non-destructive method for monitoring mould deterioration in CRP. Spectral images were acquired using a self-developed Vis/NIR imaging system equipped with 26 LED centre wavelengths. An improved Inception ResNet integrating multi-scale convolution, residual learning, and attention-based feature refinement was developed. Its performance was compared with support vector machine, XGBoost, multilayer perceptron, 1D CNN, ResNet, and the original Inception ResNet.

**Results:**

The improved Inception ResNet combined with Savitzky–Golay preprocessing achieved the best performance, with five-fold cross-validation accuracy, precision, recall, and F1 score of 96.13 ± 0.82%, 96.21 ± 0.79%, 96.13 ± 0.82%, and 96.15 ± 0.80%, respectively. On the independent external validation set, the corresponding values were 94.27 ± 0.89%, 94.39 ± 0.85%, 94.27 ± 0.89%, and 94.22 ± 0.87%. Spectral analysis showed distinct deterioration-related responses in the visible and near-infrared regions, associated with surface colour variation, moisture redistribution, and internal quality degradation.

**Discussion:**

These findings demonstrate that Vis/NIR multispectral imaging coupled with the improved Inception ResNet provides an effective and interpretable approach for rapid, non-destructive CRP quality screening and mould-deterioration monitoring.

## 1 Introduction

**Figure 1:**
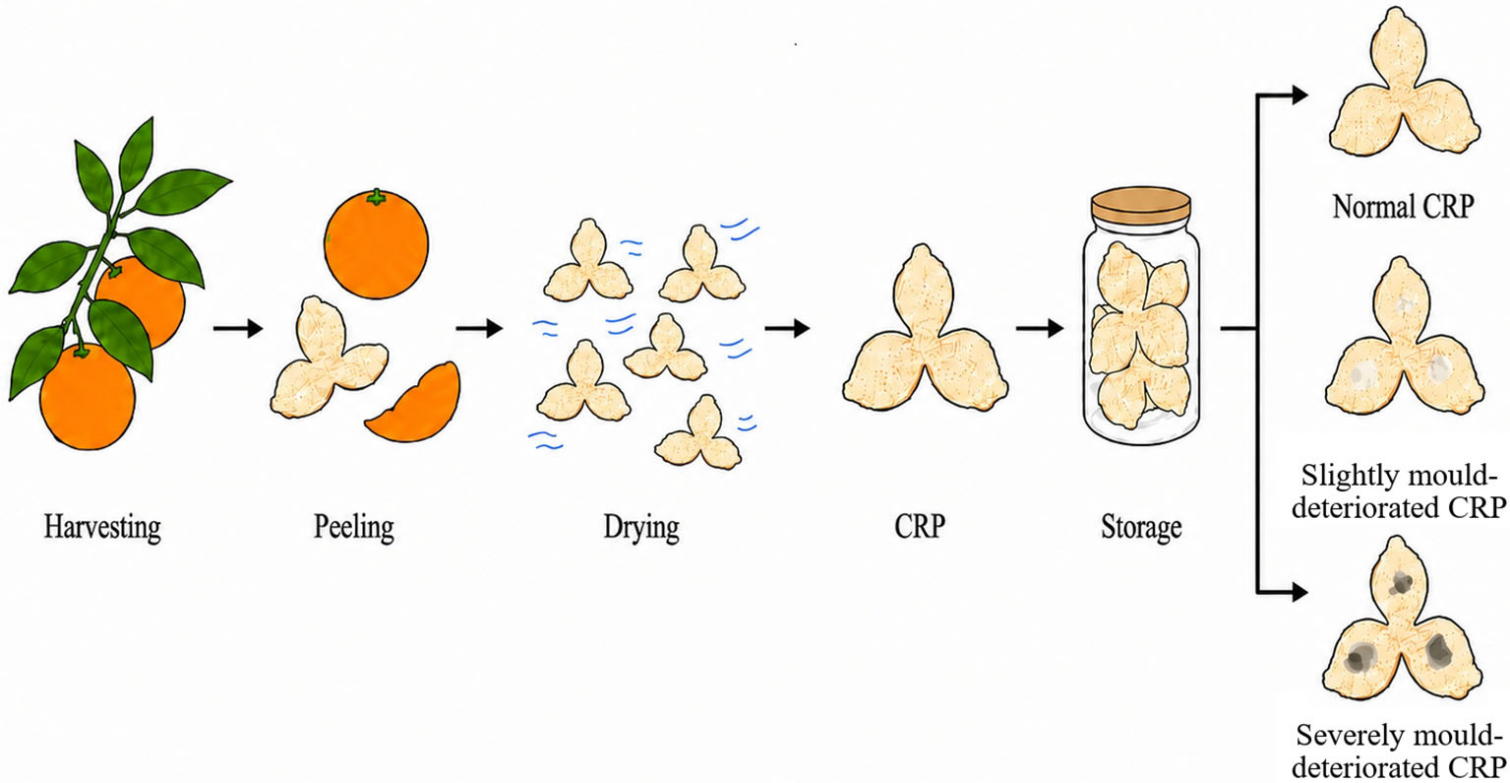
Schematic illustration of CRP processing and mould deterioration during storage.

Citri Reticulatae Pericarpium (CRP) is a representative food-medicine homologous product with wide applications in food processing, traditional dietary products, and functional ingredient development (Cai et al., 2025; Qiu et al., 2024; Hu et al., 2025). Its quality is closely associated with raw material properties, processing conditions, ageing time, and storage environment (Tang et al., 2026). During storage, CRP is often exposed to fluctuations in temperature and humidity (Tang et al., 2026; Tan et al., 2024). Improper storage may promote microbial growth and lead to mould deterioration (Tan et al., 2024; Liu et al., 2025). Such deterioration can alter the surface appearance, internal chemical composition, and sensory quality of CRP (Huang et al., 2024), thereby reducing its commercial value and raising potential safety concerns (Tan et al., 2024; Qiu et al., 2024). The processing and mould deterioration of CRP are illustrated in **Figure 1**.

At present, CRP quality control during storage still relies mainly on manual inspection and conventional laboratory analysis (Zhang et al., 2025). Visual inspection is simple and low cost, but it is subjective and has limited sensitivity, especially for early mould deterioration or samples whose visible mould has been removed before sale (Tan et al., 2024). Chemical and chromatographic methods can provide accurate information on bioactive compounds and volatile components, but they usually require sample pretreatment, chemical reagents, and relatively long testing times (Huang et al., 2024). Therefore, these methods are not well suited for rapid batch screening or online storage management. A rapid, non-destructive, and reproducible detection method is needed for CRP storage quality control (Zhang et al., 2025; Ashtiani and Martynenko, 2025).

Accordingly, spectral sensing and imaging technologies have been explored as rapid and non-destructive alternatives for food quality and safety assessment. Existing studies have demonstrated the value of Vis/NIR hyperspectral imaging, hyperspectral fluorescence imaging, and deep-learning-based spectral image analysis for nutrient quantification, fungal infection detection, aflatoxin assessment, and spectral image reconstruction in agricultural and food products (Ahmed et al., 2024; Chun et al., 2024; Pang et al., 2025; Shi et al., 2024; Yang et al., 2025; Patel et al., 2024; Miao et al., 2025); for CRP, portable NIR spectroscopy and selected wavelengths were also used to discriminate mould-damaged samples (Tan et al., 2024). These studies indicate that mould deterioration can produce measurable spectral responses, but they also reveal a practical gap for CRP storage monitoring: single-point NIR spectroscopy lacks spatial information, whereas full hyperspectral imaging usually generates large data volumes and requires more complex hardware and computation. Previous studies have noted that hyperspectral imaging is often useful for identifying informative wavelengths that can be transferred to faster multispectral systems for practical inspection (Lu and Park, 2008; Qin et al., 2013). Therefore, instead of adopting a full hyperspectral system, this study used a Vis/NIR multispectral imaging system with 26 LED centre wavelengths to capture spatial surface information and mould-sensitive spectral responses while reducing redundant data acquisition. This design offers a practical compromise for rapid CRP batch screening and provides a compact spectral sequence for the subsequent improved Inception ResNet model (Li et al., 2021).

Although Vis/NIR multispectral imaging provides compact spatial-spectral information, the spectral changes caused by CRP mould deterioration are often weak, gradual, and affected by multiple factors. Vis/NIR imaging can capture changes related to surface colour, moisture redistribution, tissue structure, and chemical composition (Jiang et al., 2025; Pang et al., 2025; Feng et al., 2025). Compared with single-point spectroscopy, it has better potential for heterogeneous samples such as CRP because both spectral and surface information can be recorded (Qin et al., 2013; Gowen et al., 2007; Feng and Sun, 2012). However, early mould deterioration may generate subtle spectral differences, and the boundary between slightly and severely deteriorated samples is not completely discrete (Tan et al., 2024; Chun et al., 2024; Pang et al., 2025). Traditional machine-learning models, such as support vector machine and XGBoost, depend strongly on manually defined feature representations and may have limited ability to describe nonlinear and multi-scale spectral patterns (Chen et al., 2026; Liu et al., 2024; Zaukuu et al., 2024). Deep learning can automatically extract hierarchical features from spectral data, but simple networks may still be insufficient for capturing mould-related responses distributed across multiple wavelength regions (Lanjewar et al., 2024; Rashvand et al., 2025; Aqeel et al., 2025).

Inception ResNet combines multi-scale convolution and residual learning, allowing features to be extracted from different receptive fields while improving information transmission in deep networks (Yu et al., 2025). This structure is suitable for spectral classification because mould deterioration may affect several wavelength regions simultaneously rather than a single isolated band (Kabir et al., 2025). Nevertheless, direct use of the original Inception ResNet may introduce redundant feature responses when applied to limited multispectral data. To address this issue, an improved Inception ResNet was developed in this study. The proposed model integrates multi-scale spectral feature extraction, residual learning, and attention-based feature refinement to enhance mould-sensitive information and suppress less informative spectral responses.

**Figure 2:**
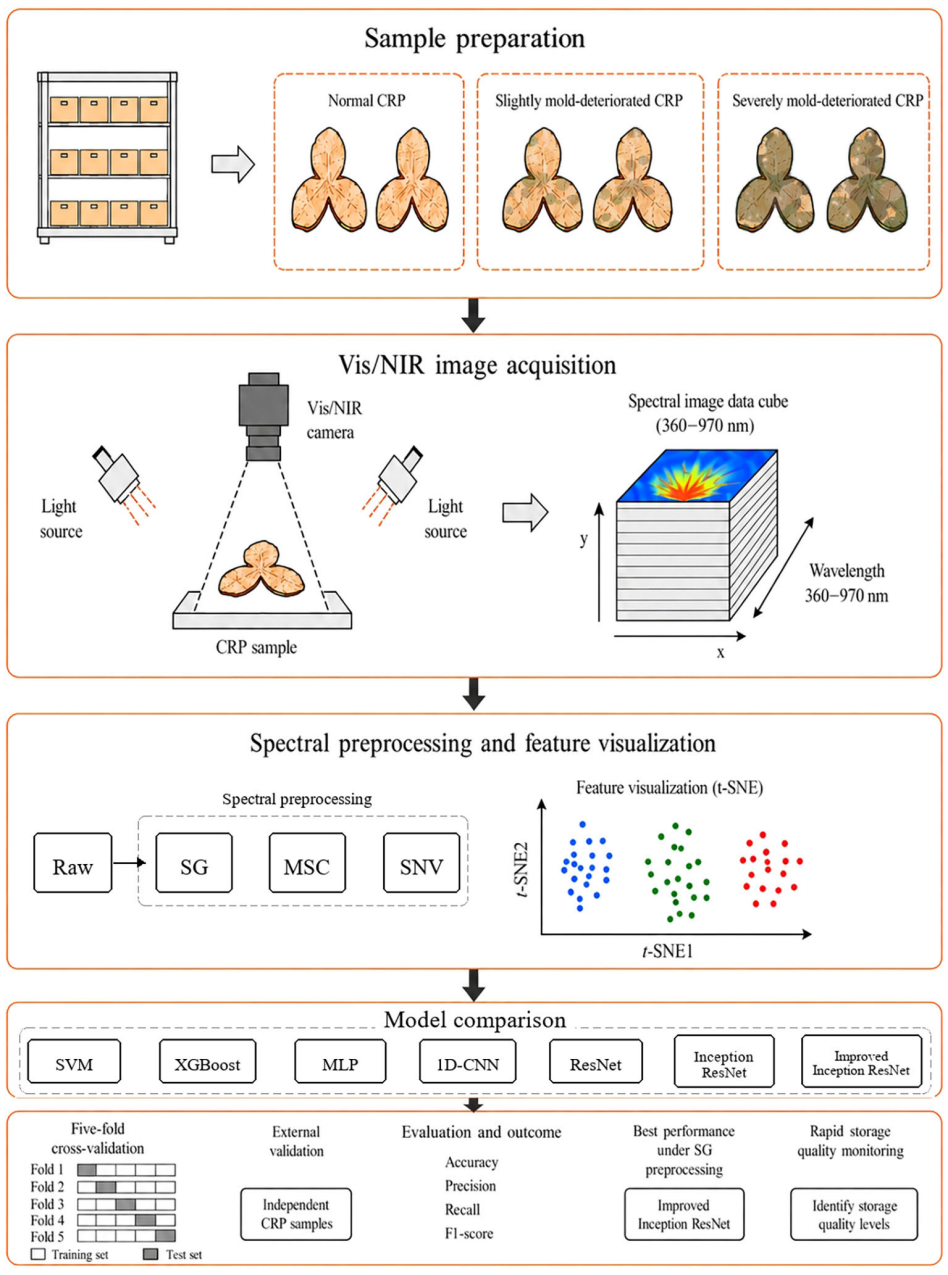
Overall workflow for CRP mould deterioration monitoring using Vis/NIR multispectral imaging and model comparison. The sample preparation panel distinguishes normal, slightly mould-deteriorated, and severely mould-deteriorated CRP, and the preprocessing methods and classification models are shown as parallel comparison branches.

The objectives of this study were therefore to develop a rapid and non-destructive Vis/NIR multispectral imaging method for CRP mould deterioration monitoring and to evaluate the effectiveness of an improved Inception ResNet for spectral classification. The main contributions are as follows. First, a Vis/NIR multispectral imaging system was applied to characterise normal, slightly mould-deteriorated, and severely mould-deteriorated CRP samples during storage. Second, an improved Inception ResNet was constructed to enhance mould-related feature extraction through multi-scale convolution, residual learning, and attention-based refinement. Third, the proposed model was systematically compared with SVM, XGBoost, MLP, 1D CNN, ResNet, and the original Inception ResNet under different preprocessing conditions, and its spectral decision mechanism was further interpreted using attribution analysis. The overall workflow of the proposed method is shown in **Figure 2**, including CRP sample preparation, Vis/NIR multispectral image acquisition, image calibration, spectral extraction, spectral preprocessing, model construction, and performance evaluation.

## 2 Materials and methods

### 2.1 CRP sample preparation

CRP samples for model development were obtained from commercial CRP suppliers in Xinhui, Jiangmen, Guangdong Province, China, a representative production area of Citri Reticulatae Pericarpium, in October 2025. Samples with complete morphology, similar sizes, and no visible mechanical damage were selected for the experiment. A total of 600 CRP samples were used for model development, including 200 normal samples, 200 slightly mould-deteriorated samples, and 200 severely mould-deteriorated samples. In addition, an independent external validation set of 150 samples was collected in December 2025, including 50 normal, 50 slightly mould-deteriorated, and 50 severely mould-deteriorated samples. The external validation samples were purchased from different commercial distributors and were not used for model training, parameter optimisation, or cross-validation.

**Figure 3:**
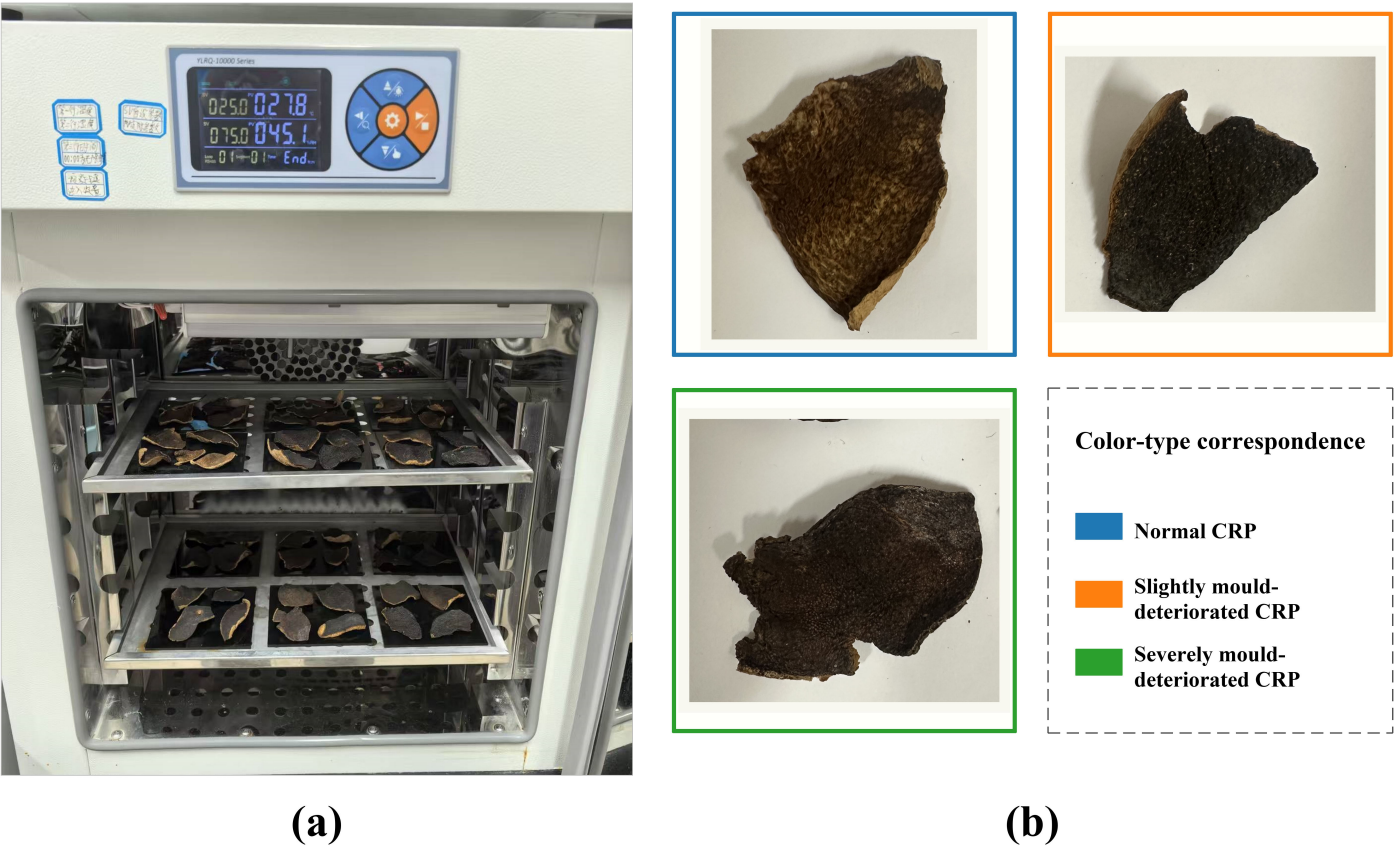
Controlled mould-deterioration treatment and representative CRP samples. **(a)** Controlled mould-deterioration treatment in a constant-temperature and humidity chamber. **(b)** Representative normal, slightly mould-deteriorated, and severely mould-deteriorated CRP samples; the frame colours correspond to the class colours used in the spectral-curve figures.

CRP samples were classified into three mould-deterioration levels based on comprehensive visual, textural, and olfactory assessment (**Table 1**). Normal samples showed no visible mould colonies and retained the characteristic orange-red or brownish-red surface, firm peel texture, and citrus aroma. Slightly mould-deteriorated samples showed scattered or localised pale grey/white mould colonies, slight colour darkening, weakened citrus aroma, and faint musty odour; the visible mould-covered area was limited to 10% of the sample surface. Severely mould-deteriorated samples showed continuous or extensive grey-green to dark mould coverage, obvious surface discolouration, softened or adhesive texture, and strong musty or putrid odour; the visible mould-covered area was greater than 30%. Samples with an ambiguous intermediate appearance were excluded or reassessed by a CRP expert before model development. Representative images are shown in **Figure 3**.

**Table 1:** Visual, textural, and olfactory criteria used for CRP mould-deterioration grading.

Class	Visual appearance	Texture and odour	Labelling rule
Normal CRP	Orange-red to brownish-red surface; no visible mould colonies	Firm peel texture and characteristic citrus aroma	Directly assigned as normal when no visible deterioration was observed
Slightly mould-deteriorated CRP	Scattered or localised pale grey/white mould colonies; slight colour darkening	Weakened citrus aroma and faint musty odour	Visible mould-covered area limited to 10% of the sample surface
Severely mould-deteriorated CRP	Continuous or extensive grey-green to dark mould coverage; obvious surface discolouration	Softened or adhesive texture and strong musty or putrid odour	Visible mould-covered area greater than 30% of the sample surface

Before spectral acquisition, each sample was gently cleaned with a soft brush to remove surface dust and loose particles. The samples were then equilibrated under the same laboratory conditions to reduce the influence of temperature fluctuations on spectral acquisition.

### 2.2 Controlled mould-deterioration treatment

To simulate mould deterioration during improper high-humidity storage, a subset of the CRP samples was placed in a constant-temperature and humidity chamber (**Figure 3**). Before mould induction, the samples were equilibrated at 23 ^°^C and 65% relative humidity (RH) for 48 h. They were then transferred to 30 ^°^C and 85% RH for up to 20 days. During the chamber treatment, the samples were spread in a single layer on clean black sample trays without overlap, placed on separate shelves to maintain comparable exposure to chamber temperature and humidity, and observed and imaged once per day at a fixed time. Samples were assigned to normal, slightly mould-deteriorated, or severely mould-deteriorated groups when the corresponding visual and sensory criteria were reached.

### 2.3 Vis/NIR multispectral imaging system

**Figure 4:**
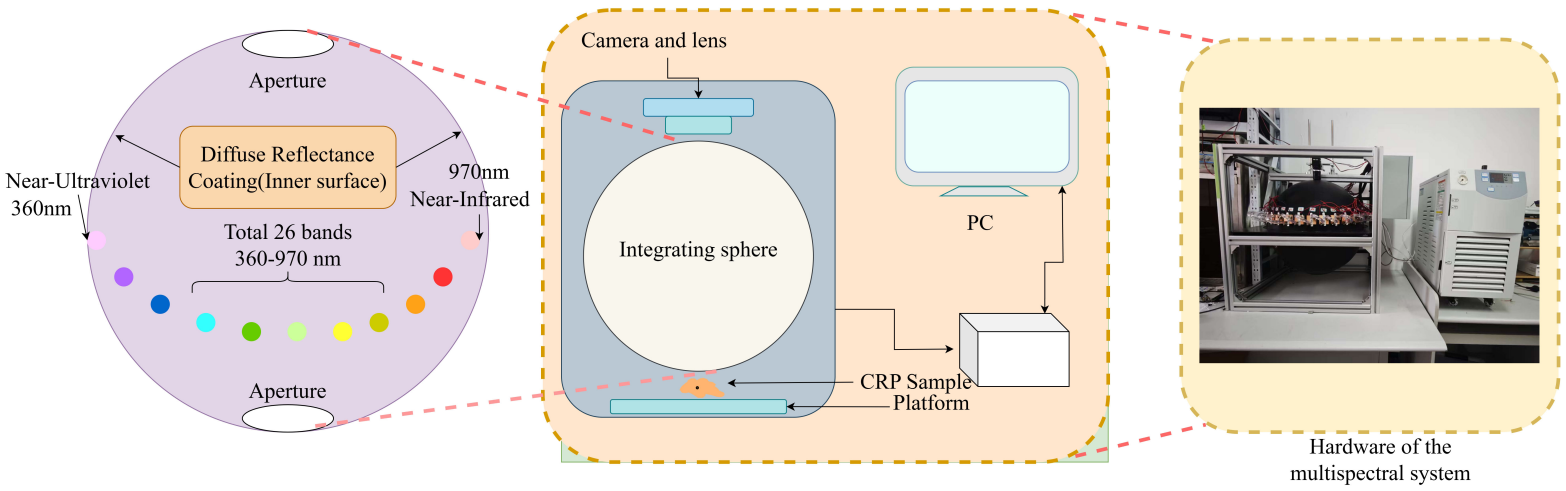
Schematic representation and hardware photograph of the self-developed Vis/NIR multispectral imaging system. The system used 26 discrete LED centre wavelengths for CRP spectral-image acquisition, and the individual centre wavelengths are listed in **Table 2**.

The Vis/NIR multispectral imaging system used in this study was self-developed by our laboratory for rapid CRP surface spectral acquisition. The system consisted of a 0.5-m Labsphere integrating sphere/diffuse reflectance module, 26 LED centre-wavelength channels, an LT665RM camera (Lumenera/Teledyne Lumenera, Canada; image resolution: 2752 × 2192 pixels; full-resolution frame rate: 27 fps), a camera-and-lens module, a moving sample platform, and a PC equipped with in-house image-acquisition software. The 26 LED centre wavelengths were 360, 380, 395, 400, 410, 420, 430, 440, 455, 465, 475, 500, 530, 570, 585, 620, 660, 700, 730, 750, 770, 805, 850, 900, 940, and 970 nm. For this LED-based system, spectral sampling was defined by the discrete LED centre wavelengths rather than by continuous hyperspectral resolution. The system schematic and hardware photograph are shown in **Figure 4**, and the system/acquisition parameters are summarised in **Table 2**.

**Table 2:** Configuration and acquisition parameters of the self-developed Vis/NIR multispectral imaging system.

Parameter	Information
System type	Self-developed Vis/NIR multispectral imaging system for CRP spectral-image acquisition
Illumination module	0.5-m Labsphere integrating sphere/diffuse reflectance module
LED centre wavelengths	360, 380, 395, 400, 410, 420, 430, 440, 455, 465, 475, 500, 530, 570, 585, 620, 660, 700, 730, 750, 770, 805, 850, 900, 940, and 970 nm
Spectral sampling	Discrete LED centre wavelengths; the system is multispectral rather than continuous hyperspectral
LED bandwidth/FWHM	Not separately measured in this study; spectral sampling was reported using the calibrated LED centre wavelengths
Camera	LT665RM camera (Lumenera/Teledyne Lumenera, Canada)
Camera resolution and frame rate	2752 × 2192 pixels; 27 fps at full resolution
Sample platform	Moving sample platform used to maintain stable sample placement during image acquisition
Exposure/integration time	Automatically determined for each wavelength according to the white-board calibration response and then kept consistent for sample acquisition
Calibration	White-reference calibration using a standard white calibration board and dark-reference calibration under the same acquisition settings
Acquisition software	In-house PC-based image-acquisition software

During image acquisition, the moving platform was used for sample positioning and operated in an intermittent mode. The platform remained stationary during image capture to ensure stable spectral acquisition. The acquisition software was developed in-house and was used to control wavelength switching, exposure/integration time determination, image acquisition, and raw image storage.

### 2.4 Image calibration and spectral extraction

White-reference calibration was performed before sample acquisition using a standard white calibration board, and the exposure/integration time for each wavelength was automatically determined according to the white-board calibration response. Dark-reference calibration was then performed by recording the dark response under the same acquisition settings. The calibrated reflectance image was calculated as shown in Equation 1:

(1) $$R=\frac{I-D}{W-D},$$

where *R* is the corrected reflectance image, *I* is the raw spectral image, *D* is the dark reference image, and *W* is the white reference image. This calibration reduced the influence of illumination non-uniformity and detector dark current.

After calibration, the region of interest (ROI) of each CRP sample was extracted from the multispectral image. Background pixels were removed by threshold segmentation. The average spectrum of all pixels in the ROI was used as the spectral vector of the sample. This operation reduced local pixel fluctuation and represented the overall spectral characteristics of each CRP sample.

### 2.5 Spectral preprocessing

Spectral preprocessing was performed to reduce noise, correct baseline drift, and minimise scattering effects. It should be noted that spectral preprocessing is equally beneficial for multispectral data, as random noise, baseline drift, and surface scattering are universal measurement artefacts independent of spectral dimensionality. Three preprocessing methods were used in this study: Savitzky-Golay (SG) smoothing, multiplicative scatter correction (MSC), and standard normal variate (SNV).

For spectral preprocessing, SG smoothing was applied with a window length of 11 and polynomial order of 3. MSC was used as a scatter-correction procedure. For each cross-validation split, the reference spectrum for MSC was calculated only from the training fold and then applied to the corresponding validation/test fold; thus, the validation/test spectra did not participate in estimating the MSC reference. SNV was applied independently to each sample spectrum by subtracting the mean of that sample spectrum and dividing by its standard deviation. Therefore, SNV did not use information from other samples or from the validation/test set.

### 2.6 Improved inception ResNet

To improve the recognition of mould deterioration in CRP, an improved Inception ResNet was developed for Vis/NIR spectral data. The input of the network was the spectral vector extracted from the region of interest. Let the input spectrum be denoted as **x** ∈ R*^N^*, where *N* represents the number of spectral bands. The proposed network aimed to enhance mould-related spectral information through multi-scale feature extraction, residual learning, and attention-based feature refinement.

The original Inception ResNet combines parallel convolution branches and residual connections. This structure is suitable for spectral classification because different wavelength regions may contain complementary information. However, mould deterioration in CRP may cause weak spectral changes across several bands. The direct use of the original Inception ResNet may introduce redundant features and reduce the sensitivity to mould-related responses. Therefore, three modifications were introduced in this study.

First, a multi-scale convolution module was designed. Convolution kernels with different sizes were used to extract spectral features at different receptive fields. Small kernels captured local spectral fluctuations, while larger kernels extracted broader spectral trends. The multi-scale feature extraction process was expressed as shown in Equation 2:

(2) $$F_{ms}=\text{Concat}\left[{\text{Conv}}_{1\times 3}\left(x\right),{\text{Conv}}_{1\times 5}\left(x\right),{\text{Conv}}_{1\times 7}\left(x\right)\right],$$

where Conv_1×3_, Conv_1×5_, and Conv_1×7_ denote convolution operations with kernel sizes of 3, 5, and 7, respectively. Concat[·] denotes feature concatenation along the channel dimension.

A residual connection was introduced to improve feature transmission and reduce the risk of gradient degradation. Since the number of channels may change after feature concatenation, a 1 × 1 convolution was used to match the feature dimension when necessary. The residual output was calculated as shown in Equation 3:

(3) $$F_{res}=F_{ms}+\phi \left(x\right),$$

where *ϕ*(·) denotes the dimension matching operation. When the input and output dimensions were identical, *ϕ*(**x**) was an identity mapping. Otherwise, *ϕ*(**x**) was implemented by a 1 × 1 convolution.

An efficient channel attention module was embedded after the residual block. This module was used to adaptively assign weights to different spectral feature channels. It enhanced informative features related to mould deterioration and suppressed less useful information. The attention weight was calculated as shown in Equations 4–6:

(4) $$z=\text{GAP}\left(F_{res}\right),$$

(5) $$a=\sigma \left(\text{Conv}1{\text{D}}_{k}\left(z\right)\right),$$

(6) $$F_{att}=F_{res}⊗a,$$

where GAP(·) denotes global average pooling, Conv1D*_k_*(·) denotes a convolution operation with kernel size *k*, *σ*(·) is the sigmoid activation function, and ⊗ denotes channel wise multiplication. **F***_att_* is the refined feature output.

The classification module consisted of global average pooling, dropout, and a fully connected layer. The softmax function was used to obtain the probability of each class as shown in Equation 7:

(7) $$\hat{y}=\text{Softmax}\left(Wh+b\right),$$

where **h** is the feature vector after global average pooling, **W** and **b** are trainable parameters, and $\hat{y}$ is the predicted class probability.

The overall structure of the improved Inception ResNet is shown in **Table 3**. Batch normalisation and ReLU activation were used after each convolution layer to improve training stability and nonlinear representation.

**Table 3:** Structure of the improved Inception ResNet for CRP mould deterioration detection.

Layer	Output size	Kernel size	Operation
Input	1 × *N*	–	Spectral vector
Initial Conv	32 × *N*	1 × 3	Conv1D + BN + ReLU
Inception branch 1	32 × *N*	1 × 3	Conv1D + BN + ReLU
Inception branch 2	32 × *N*	1 × 5	Conv1D + BN + ReLU
Inception branch 3	32 × *N*	1 × 7	Conv1D + BN + ReLU
Concatenation	96 × *N*	–	Multi-scale feature fusion
Dimension matching	96 × *N*	1 × 1	Conv1D
Residual connection	96 × *N*	–	Feature addition
Attention module	96 × *N*	–	Channel attention refinement
Global pooling	96 × 1	–	Global average pooling
Dropout	96 × 1	–	Dropout regularisation
Fully connected	*C*	–	Dense layer
Output	*C*	–	Softmax classification

In this study, the improved Inception ResNet was trained using the cross entropy loss function as shown in Equation 8:

(8) $$ℒ=-\frac{1}{M}\sum_{i=1}^{M}\sum_{c=1}^{C}y_{ic}\text{log }({\hat{y}}_{ic}),$$

where *M* is the number of training samples, *C* is the number of classes, *y_ic_*is the true label, and ${\hat{y}}_{ic}$ is the predicted probability. The Adam optimizer was used for parameter updating. Early stopping was adopted to reduce overfitting. The proposed network was compared with SVM, XGBoost, MLP, 1D CNN, ResNet, and the original Inception ResNet under the same data division and evaluation strategy. The complete layer-wise structure of the proposed network is summarised in **Table 3**.

### 2.7 Comparative models

To evaluate the effectiveness of the proposed model, six comparative models were established. These models included support vector machine (SVM), XGBoost, multilayer perceptron (MLP), 1D CNN, ResNet, and the original Inception ResNet. SVM and XGBoost were used as representative traditional machine-learning models. MLP was used to evaluate the performance of a fully connected neural network on spectral data. A 1D CNN was used to extract local spectral features. ResNet was used to examine the effect of residual learning. The original Inception ResNet was used as the baseline architecture for comparison with the improved model.

For traditional machine-learning models, the optimised parameters were limited to model training hyperparameters. For SVM, the candidate kernel types were RBF and linear, and the selected kernel was RBF. The penalty parameter *C* was searched over {0.01,0.1,1,10,100}, and *γ* was searched over {scale,0.001,0.01,0.1,1}. The selected values were *C* = 10 and *γ* = scale. For XGBoost, the search ranges were *n_estimators* = {100,200,300}, *max_depth* = {3,4,5,6}, *learning_rate* = {0.01,0.05,0.10}, *subsample* = {0.8,1.0}, *colsample_bytree* = {0.8,1.0}, *min_child_weight* = {1,3,5}, and *reg_lambda* = {1,5,10}. The selected XGBoost values were *n_estimators* = 200, *max_depth* = 4, *learning_rate* = 0.05, *subsample* = 0.8, *colsample_bytree* = 0.8, *min_child_weight* = 1, and *reg_lambda* = 1. All these settings were selected using only the training folds.

For deep-learning models, including MLP, 1D CNN, ResNet, the original Inception ResNet, and the proposed improved Inception ResNet, the essential training settings are reported in **Table 4**. These include an input dimension of 26, 3 output classes, the Adam optimizer, an initial learning rate of 0.001, a batch size of 32, and a maximum of 400 epochs. Model-architecture selection and training-parameter adjustment were conducted using only the training folds. The held-out test folds and the independent external validation set were reserved for final evaluation and were not used for preprocessing fitting, hyperparameter tuning, early stopping, or model selection, thereby reducing the risk of data leakage.

**Table 4:** Summary of model training and validation settings.

Item	Information
Input dimension	26 wavelength variables
Output classes	3 classes: normal, slightly mould-deteriorated, and severely mould-deteriorated CRP
Deep-learning optimizer	Adam
Initial learning rate	0.001
Batch size	32
Maximum epochs	400
Data-leakage prevention	Preprocessing fitting, parameter selection, early stopping, and model selection were performed only within the training folds; test folds and the external validation set were reserved for final evaluation

### 2.8 Model training and validation

Five-fold cross-validation was used to evaluate model stability. In each fold, 80% of the internal dataset was used for training and 20% was used for testing. Therefore, 480 samples were used for training and 120 samples were used for testing in each fold. The process was repeated five times, and the mean performance and standard deviation were reported.

An independent external validation set was used to further assess the generalisation ability of the models. This set contained 150 samples, including 50 normal samples, 50 slightly mould-deteriorated samples, and 50 severely mould-deteriorated samples. The external validation samples represented an independent batch relative to the model-development set and were not involved in model training, parameter optimisation, preprocessing fitting, cross-validation, early stopping, or model selection. The sample type/variety information, origin, sampling date, class composition, and batch independence of the external validation set are summarised in **Table 5**.

**Table 5:** Basic information of the independent external validation samples.

Class	Number	Origin and sampling date	Independence and treatment information
Normal CRP	50	Xinhui, Jiangmen, Guangdong Province, China; December 2025	Different commercial distributors from the model-development set; not used for training, tuning, or cross-validation
Slightly mould-deteriorated CRP	50	Xinhui, Jiangmen, Guangdong Province, China; December 2025	Same chamber treatment and visual/sensory grading criteria as model-development samples
Severely mould-deteriorated CRP	50	Xinhui, Jiangmen, Guangdong Province, China; December 2025	Same chamber treatment and visual/sensory grading criteria as model-development samples

All experiments were conducted on a PC with an AMD Ryzen 9 9950X processor, an NVIDIA GeForce RTX 5070 Ti graphics card, and 32 GB of RAM. The software environment was based on Python 3.12.4. Spectral preprocessing, model construction, training, and validation were implemented under the same computational environment to ensure consistency and reproducibility of the experimental results.

### 2.9 Evaluation metrics

The classification performance was evaluated using accuracy, precision, recall, and F1 score. These metrics were calculated as shown in Equations 9–12:

(9) $$Accuracy=\frac{TP+TN}{TP+TN+FP+FN}$$

(10) $$Precision=\frac{TP}{TP+FP}$$

(11) $$Recall=\frac{TP}{TP+FN}$$

(12) $$F1=\frac{2\times Precision\times Recall}{Precision+Recall}$$

where *TP*, *TN*, *FP*, and *FN* represent true positive, true negative, false positive, and false negative samples, respectively.

For five-fold cross-validation, the final performance was expressed as mean ± standard deviation. The confusion matrix was also used to analyse the classification errors between normal and mould-deteriorated CRP samples.

### 2.10 Explainability analysis

To further understand the decision mechanism of the proposed improved Inception ResNet, an explainability analysis was conducted on the SG-preprocessed spectra. Three complementary attribution strategies were used, including 1D Grad-CAM, integrated gradients, and attention-weight visualisation. The three attribution maps were then integrated to obtain a unified wavelength-level importance score.

For the *j*-th wavelength *λ_j_*(*j* = 1,2*,…,N*), the raw attribution score obtained from method *m* was denoted as *s_m,j_*, where *m* ∈ {*GC,IG,ATT*} represents 1D Grad-CAM, integrated gradients, and attention weights, respectively. To make the three attribution maps comparable, each score was first normalised by min-max normalisation as shown in Equation 13:

(13) $${\tilde{s}}_{m,j}=\frac{s_{m,j}-\min_{j}(s_{m,j})}{\max_{j}(s_{m,j})-\min_{j}(s_{m,j})+\epsilon },$$

where *ϵ* is a small constant used to avoid division by zero. The integrated importance score *I_j_*was then calculated by equal-weight fusion of the three normalised attribution scores as shown in Equation 14:

(14) $$I_{j}=\frac{1}{3}\left({\tilde{s}}_{GC,j}+{\tilde{s}}_{IG,j}+{\tilde{s}}_{ATT,j}\right).$$

Wavelengths with higher *I_j_*values were considered to contribute more strongly to the classification of CRP mould deterioration. The high-importance centre wavelengths were further interpreted according to known Vis/NIR responses related to colour variation, moisture redistribution, surface scattering, and internal quality deterioration.

## 3 Results and discussion

### 3.1 Spectral response analysis of CRP samples

**Figure 5:**
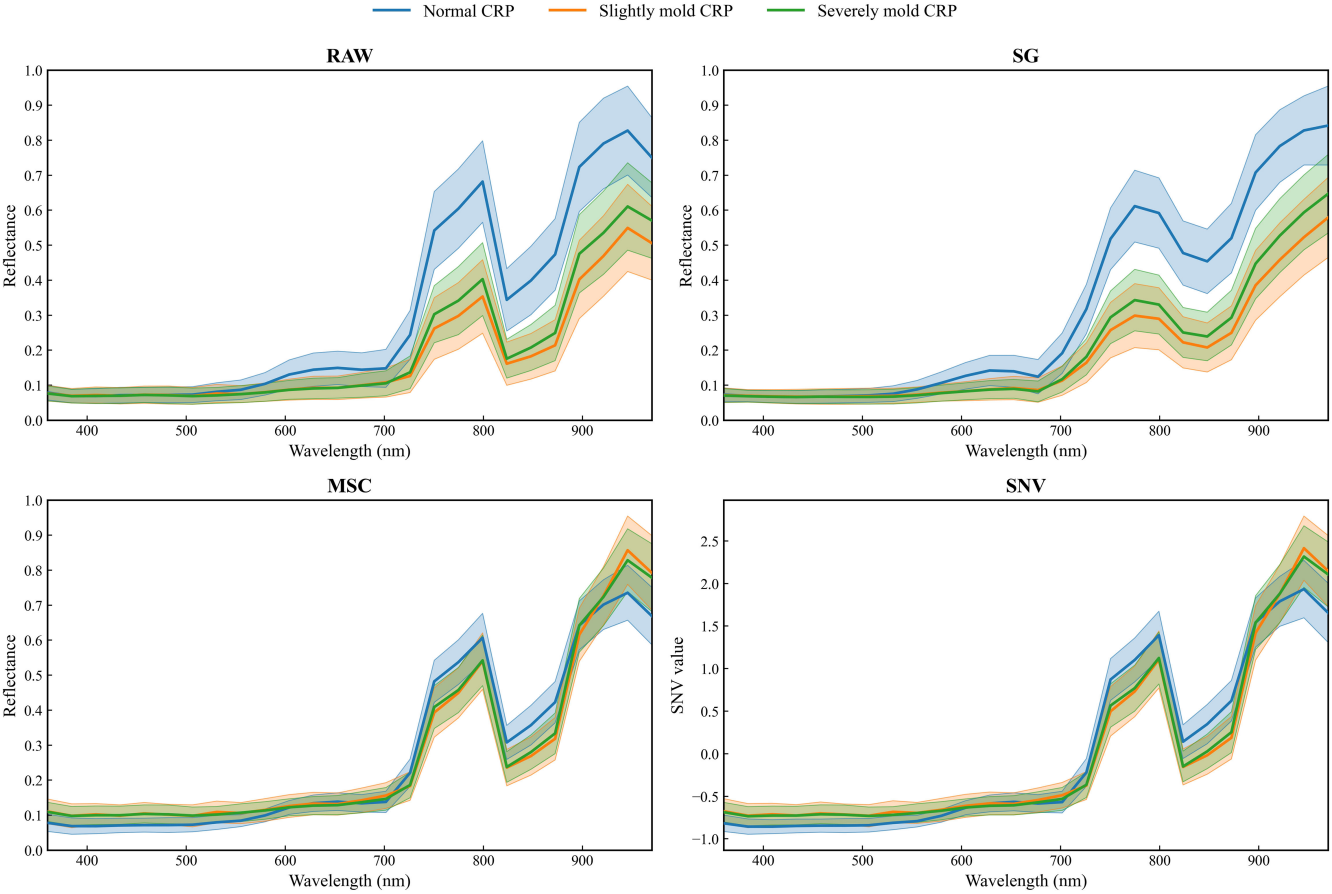
Mean spectra of normal CRP, slightly mould-deteriorated CRP, and severely mould-deteriorated CRP at the 26 LED centre wavelengths.

The mean spectral curves of normal CRP, slightly mould-deteriorated CRP, and severely mould-deteriorated CRP are shown in **Figure 5**. Because the self-developed system used 26 LED centre wavelengths, the curves represent discrete multispectral measurements rather than continuous hyperspectral spectra. The three groups showed similar overall spectral trends across the 26 centre wavelengths, but clear differences were observed in several wavelength regions.

In the visible region, differences among the three groups were mainly related to surface colour, pigment-related reflectance, and fungal coverage. These responses are consistent with previous Vis/NIR studies showing that visible wavelengths are sensitive to surface appearance and colour-related quality changes (Grassi et al., 2021). More obvious spectral differences appeared in the near-infrared region. Normal CRP generally showed higher reflectance than mould-deteriorated samples, especially after 730 nm. The difference was particularly clear around the available centre wavelengths of 730, 750, 770, 805, 900, 940, and 970 nm. These centre wavelengths are usually associated with moisture redistribution, tissue/scattering variation, and O–H or C–H related overtone/combination absorption responses (Grassi et al., 2021; Zhang et al., 2024). Previous CRP-specific NIR work also confirmed that mould-damaged CRP can produce discriminative spectral differences (Tan et al., 2024). Therefore, the spectral changes may be related to increased surface roughness, fungal coverage, water-state variation, and internal quality deterioration during mould development (Tan et al., 2024; Pang et al., 2025).

After SG preprocessing, random fluctuations in the spectra were reduced, and the spectral profiles became smoother. The relative differences among the three classes were still retained. This indicates that SG preprocessing effectively reduced high-frequency noise without removing useful mould-related spectral information. After MSC and SNV preprocessing, the influence of scattering and intensity variation was reduced. However, the differences between slightly and severely mould-deteriorated CRP became less stable in some wavelength regions. This suggests that part of the discriminative information may be related to surface scattering caused by mould growth. Excessive scatter correction may weaken these useful differences.

Mould deterioration caused detectable spectral changes in CRP. The separation between normal and mould-deteriorated samples was more pronounced than that between slightly and severely mould-deteriorated samples. This finding is consistent with the gradual nature of mould development. Slightly and severely mould-deteriorated CRP may share similar spectral responses in transitional regions, which increases the difficulty of fine-grained classification.

### 3.2 Visualisation analysis using t-SNE

**Figure 6:**
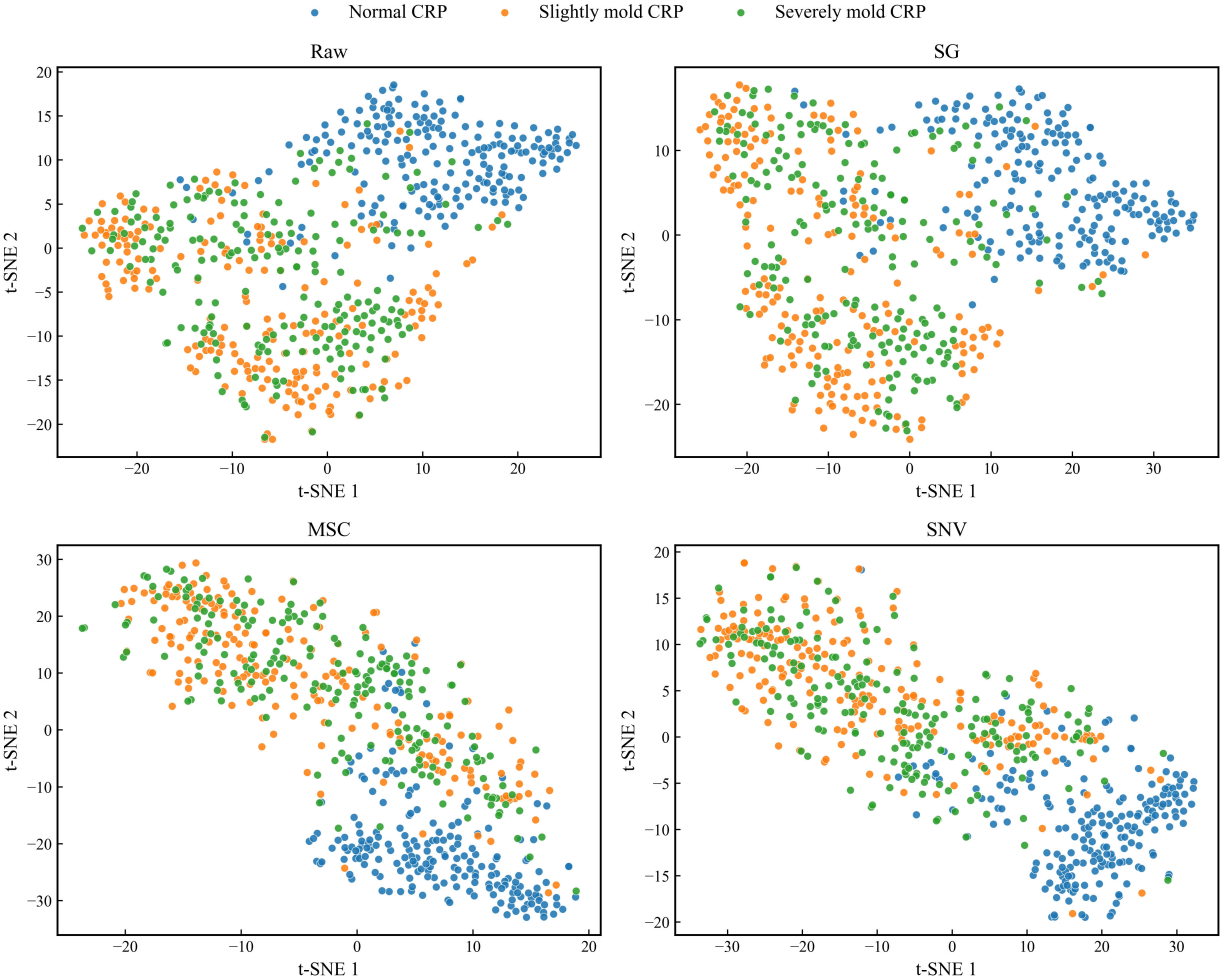
t-SNE visualisation of CRP spectra under different preprocessing methods.

The t-SNE visualisation results of raw, SG, MSC, and SNV spectra are shown in **Figure 6**. The raw spectra showed a certain degree of clustering. Normal CRP samples were mainly distributed on one side of the feature space, while slightly and severely mould-deteriorated samples were more scattered. However, the boundaries among the three classes were not sufficiently clear, especially between the two mould-deteriorated groups.

After SG preprocessing, the class distribution became more compact. Normal CRP samples formed a relatively independent cluster, while mould-deteriorated samples were mainly distributed in another region. This indicates that SG smoothing improved the consistency of spectral features and enhanced the separability of normal and mould-deteriorated CRP. However, partial overlap between slightly and severely mould-deteriorated samples remained. This overlap reflects the continuous progression of mould deterioration rather than a completely discrete change.

For MSC preprocessing, the normal samples still showed partial separation, but the distributions of slightly and severely mould-deteriorated samples showed more substantial overlap. This result suggests that scattering information may contain useful features related to mould growth on the CRP surface. When MSC removed part of the scattering effect, the differences among mould-deterioration degrees were reduced.

SNV preprocessing also improved the global distribution of samples. Normal CRP samples were clearly separated from most mould-deteriorated samples. However, slightly and severely mould-deteriorated samples still formed a continuous distribution. This indicates that SNV was effective in reducing spectral offset and intensity variation, but it could not completely distinguish different mould deterioration degrees.

**Figures 5**, **6** visually indicated the advantage of SG preprocessing. In **Figure 5**, SG smoothing reduced local spectral noise while preserving the main spectral profiles and mould-related differences. In **Figure 6**, SG preprocessing produced a more compact sample distribution and clearer separation between normal and mould-deteriorated CRP. This visual observation was further supported by the five-fold cross-validation results in **Table 6**.

**Table 6:** Five-fold cross-validation results of models under different preprocessing methods.

Pre	Model	Acc (%)	Prec (%)	Rec (%)	F1 (%)
Raw	SVM	86.42 ± 1.74	86.58 ± 1.69	86.42 ± 1.74	86.37 ± 1.71
Raw	XGBoost	87.36 ± 1.52	87.49 ± 1.48	87.36 ± 1.52	87.40 ± 1.50
Raw	MLP	88.17 ± 1.63	88.32 ± 1.58	88.17 ± 1.63	88.21 ± 1.60
Raw	1D CNN	90.58 ± 1.35	90.71 ± 1.31	90.58 ± 1.35	90.61 ± 1.33
Raw	ResNet	91.47 ± 1.28	91.62 ± 1.22	91.47 ± 1.28	91.51 ± 1.25
Raw	Inception ResNet	92.64 ± 1.16	92.76 ± 1.11	92.64 ± 1.16	92.68 ± 1.13
Raw	Improved Inception ResNet	94.18 ± 1.08	94.31 ± 1.03	94.18 ± 1.08	94.22 ± 1.05
SG	SVM	88.73 ± 1.45	88.94 ± 1.39	88.73 ± 1.45	88.78 ± 1.42
SG	XGBoost	90.16 ± 1.37	90.29 ± 1.34	90.16 ± 1.37	90.19 ± 1.35
SG	MLP	91.08 ± 1.24	91.25 ± 1.19	91.08 ± 1.24	91.12 ± 1.21
SG	1D CNN	93.27 ± 1.06	93.36 ± 1.03	93.27 ± 1.06	93.30 ± 1.04
SG	ResNet	94.05 ± 0.97	94.18 ± 0.92	94.05 ± 0.97	94.09 ± 0.94
SG	Inception ResNet	94.91 ± 0.91	95.03 ± 0.88	94.91 ± 0.91	94.94 ± 0.89
SG	Improved Inception ResNet	96.13 ± 0.82	96.21 ± 0.79	96.13 ± 0.82	96.15 ± 0.80
MSC	SVM	84.96 ± 1.91	85.24 ± 1.85	84.96 ± 1.91	84.89 ± 1.88
MSC	XGBoost	86.57 ± 1.68	86.73 ± 1.63	86.57 ± 1.68	86.61 ± 1.65
MSC	MLP	87.42 ± 1.59	87.68 ± 1.54	87.42 ± 1.59	87.47 ± 1.56
MSC	1D CNN	90.36 ± 1.42	90.49 ± 1.37	90.36 ± 1.42	90.39 ± 1.39
MSC	ResNet	91.18 ± 1.29	91.35 ± 1.24	91.18 ± 1.29	91.22 ± 1.26
MSC	Inception ResNet	92.07 ± 1.21	92.24 ± 1.16	92.07 ± 1.21	92.11 ± 1.18
MSC	Improved Inception ResNet	93.52 ± 1.04	93.69 ± 1.00	93.52 ± 1.04	93.57 ± 1.02
SNV	SVM	87.91 ± 1.56	88.13 ± 1.51	87.91 ± 1.56	87.95 ± 1.53
SNV	XGBoost	89.24 ± 1.46	89.41 ± 1.42	89.24 ± 1.46	89.28 ± 1.44
SNV	MLP	90.36 ± 1.32	90.52 ± 1.28	90.36 ± 1.32	90.40 ± 1.30
SNV	1D CNN	92.48 ± 1.13	92.63 ± 1.09	92.48 ± 1.13	92.52 ± 1.10
SNV	ResNet	93.16 ± 1.02	93.29 ± 0.98	93.16 ± 1.02	93.20 ± 1.00
SNV	Inception ResNet	94.28 ± 0.94	94.41 ± 0.90	94.28 ± 0.94	94.32 ± 0.92
SNV	Improved Inception ResNet	95.24 ± 0.88	95.37 ± 0.84	95.24 ± 0.88	95.28 ± 0.86

### 3.3 Classification performance under different preprocessing methods

**Figure 7:**
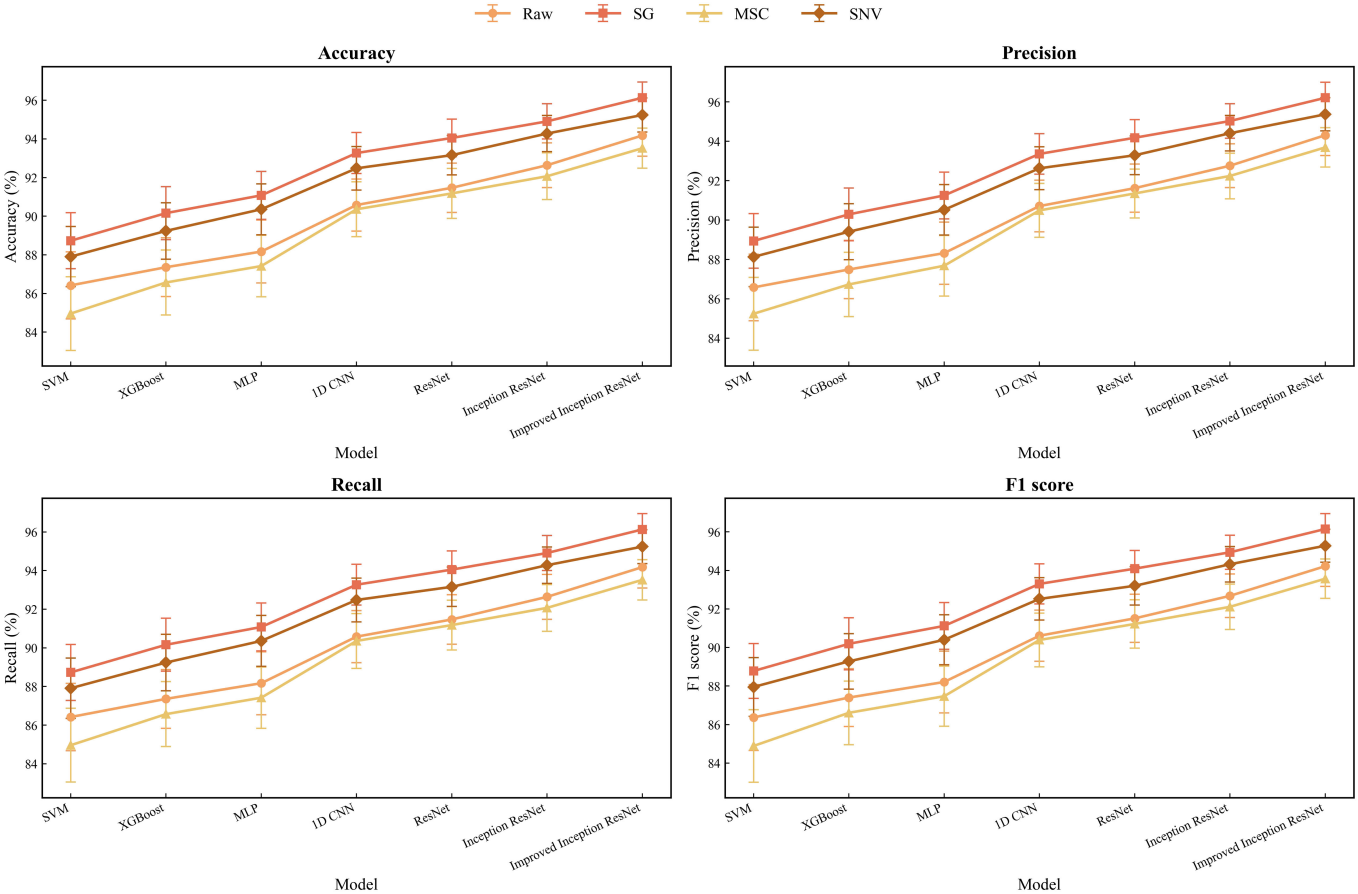
Performance comparison of different classification models under different preprocessing methods.

The classification performance of different models under raw, SG, MSC, and SNV spectra is shown in **Table 6**. The performance trends of all models under different preprocessing methods are visualised in **Figure 7**. Accuracy, precision, recall, and F1 score showed consistent trends across the tested models.

For raw spectra, traditional machine-learning models achieved moderate classification performance. SVM and XGBoost obtained accuracies of 86.42% and 87.36%, respectively. MLP achieved slightly higher accuracy than the traditional models, but its improvement was limited, probably because MLP does not explicitly extract local spectral structures. Deep-learning models showed better performance. The original Inception ResNet achieved an accuracy of 92.64%, while the improved Inception ResNet achieved 94.18%.

After SG preprocessing, the performance of most models improved. The improved Inception ResNet achieved the highest accuracy (96.13 ± 0.82%) and F1 score (96.15 ± 0.80%) under SG preprocessing, outperforming raw spectra, MSC, and SNV. This result indicates that SG preprocessing retained important spectral features while reducing noise interference. It also agrees with the t-SNE results, where SG produced clearer class separation. All performance values are expressed as mean ± standard deviation.

MSC preprocessing produced lower performance than SG and SNV for most models. The improved Inception ResNet achieved an accuracy of 93.52% under MSC preprocessing. This result may be attributed to the fact that mould growth changes both chemical absorption and surface scattering characteristics. MSC can correct scattering variation, but it may also remove part of the mould-related surface information. Therefore, its performance was lower. SNV preprocessing achieved better results than MSC but slightly lower performance than SG. The improved Inception ResNet achieved an accuracy of 95.24% under SNV preprocessing. This suggests that SNV effectively reduced spectral intensity differences, but the normalisation process may also reduce some class-specific amplitude information.

### 3.4 Comparison of different classification models

The overall comparison showed that deep-learning models outperformed traditional machine-learning models. SVM and XGBoost achieved acceptable results, but their performance was limited by their dependence on manually defined spectral representations. MLP showed slightly improved performance, but it lacked local convolutional feature extraction. Therefore, it was not sufficient to fully describe local absorption changes caused by mould deterioration.

The 1D CNN achieved better classification performance by extracting local spectral features. ResNet further improved the results through residual learning, which enhanced feature propagation and model stability. The original Inception ResNet achieved higher accuracy than ResNet because its multi-branch structure captured features at different spectral scales. However, its feature representation still contained redundant information.

The improved Inception ResNet achieved the best performance among all models. Under SG preprocessing, it improved accuracy by 1.22 percentage points compared with the original Inception ResNet. The improvement was not excessive, but it was consistent across accuracy, precision, recall, and F1 score. This indicates that the proposed multi-scale attention refinement provided stable performance gains rather than an accidental improvement.

**Figure 8:**
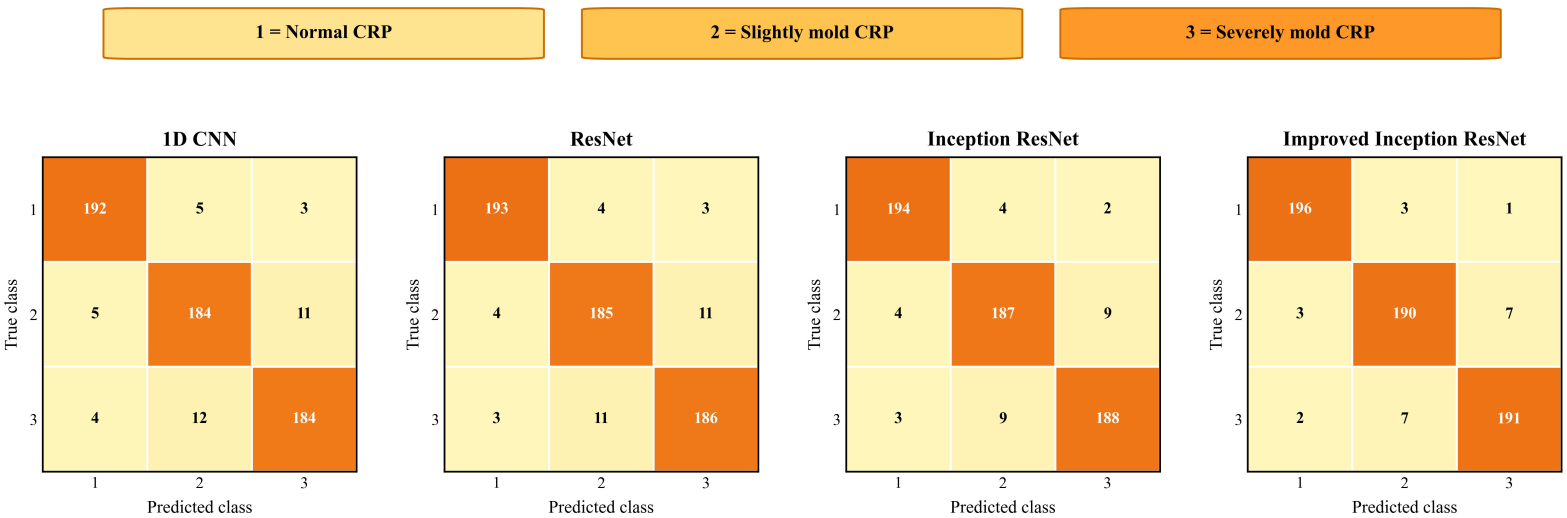
Confusion matrix of the proposed model for CRP mould deterioration classification.

The confusion matrix is presented in **Figure 8**. The confusion analysis showed that most normal CRP samples were correctly identified. The main misclassification occurred between slightly mould-deteriorated and severely mould-deteriorated samples. This result is reasonable because mould deterioration is a gradual process. The boundary between slight and severe deterioration is not completely sharp in spectral space. Some slightly mould-deteriorated samples with relatively strong fungal coverage may show spectral responses similar to severe deterioration. Conversely, some severely deteriorated samples with uneven fungal distribution may be classified as slight deterioration.

### 3.5 Ablation study of the improved inception ResNet

To verify the contribution of each module, an ablation study was conducted under SG preprocessing. The results are shown in **Table 7**. The original Inception ResNet achieved an accuracy of 94.91%. After introducing the multi-scale convolution refinement, the accuracy increased to 95.34%. This suggests that convolution kernels with different receptive fields helped extract complementary spectral features.

**Table 7:** Ablation results of the improved Inception ResNet under SG preprocessing.

Model structure	Accuracy (%)	Precision (%)	Recall (%)	F1 score (%)
Original Inception ResNet	94.91 ± 0.91	95.03 ± 0.88	94.91 ± 0.91	94.94 ± 0.89
Without attention module	95.34 ± 0.87	95.46 ± 0.83	95.34 ± 0.87	95.37 ± 0.85
Without multi-scale refinement	95.47 ± 0.92	95.58 ± 0.89	95.47 ± 0.92	95.50 ± 0.90
Improved Inception ResNet	96.13 ± 0.82	96.21 ± 0.79	96.13 ± 0.82	96.15 ± 0.80

When the attention module was added alone, the accuracy reached 95.47%. This indicates that adaptive channel weighting improved the model’s sensitivity to mould-related spectral features. The full improved Inception ResNet achieved the highest accuracy of 96.13%. The F1 score also increased to 96.15%. These results confirm that multi-scale feature extraction and attention-based feature refinement contributed jointly to the final performance.

### 3.6 External validation

An independent external validation set was used to evaluate the generalisation ability of different models. The external validation set contained 150 samples, including 50 normal CRP samples, 50 slightly mould-deteriorated CRP samples, and 50 severely mould-deteriorated CRP samples. These samples represented an independent batch relative to the model-development set and were not used for model training, parameter optimisation, preprocessing fitting, or cross-validation. The external validation samples were also obtained from Xinhui, Jiangmen, Guangdong Province, China, but they were purchased from different commercial distributors in December 2025, two months later than the October 2025 model-development sample collection. The sample type/variety information, origin, sampling date, class composition, and batch independence of the external validation set are summarised in **Table 5**.

Compared with the internal cross-validation results, the external validation performance decreased slightly. This is expected because the external samples were collected independently and were not involved in model training or parameter optimisation. The traditional machine-learning models showed a larger performance drop. For example, SVM achieved an external validation accuracy of 86.27%, while XGBoost achieved 87.60%. Deep-learning models showed better robustness. The improved Inception ResNet achieved the best external validation accuracy of 94.27 ± 0.89%, with an F1 score of 94.22 ± 0.87%.

The external validation results in **Table 8** further confirmed that the proposed model had good preliminary generalisation ability under the current source and storage-condition settings. The performance decrease from internal cross-validation to external validation was limited, indicating that the model did not rely only on sample-specific patterns. The remaining classification errors mainly occurred between slightly and severely mould-deteriorated CRP samples. This again suggests that the spectral boundary between different mould-deterioration degrees is continuous and partially overlapping. Therefore, the external validation set should be regarded as an independent-batch validation set from different distributors within the same representative production area rather than a multi-geographical-origin validation set.

**Table 8:** External validation results of different models under SG preprocessing.

Model	Accuracy (%)	Precision (%)	Recall (%)	F1 score (%)
SVM	86.27 ± 1.38	86.61 ± 1.32	86.27 ± 1.38	86.20 ± 1.35
XGBoost	87.60 ± 1.21	87.88 ± 1.17	87.60 ± 1.21	87.64 ± 1.18
MLP	88.93 ± 1.34	89.17 ± 1.29	88.93 ± 1.34	88.97 ± 1.31
1D CNN	91.07 ± 1.16	91.24 ± 1.11	91.07 ± 1.16	91.10 ± 1.13
ResNet	92.13 ± 1.05	92.36 ± 0.99	92.13 ± 1.05	92.18 ± 1.02
Inception ResNet	93.20 ± 0.96	93.38 ± 0.91	93.20 ± 0.96	93.23 ± 0.94
Improved Inception ResNet	94.27 ± 0.89	94.39 ± 0.85	94.27 ± 0.89	94.22 ± 0.87

### 3.7 Explainable spectral attribution analysis

**Figure 9:**
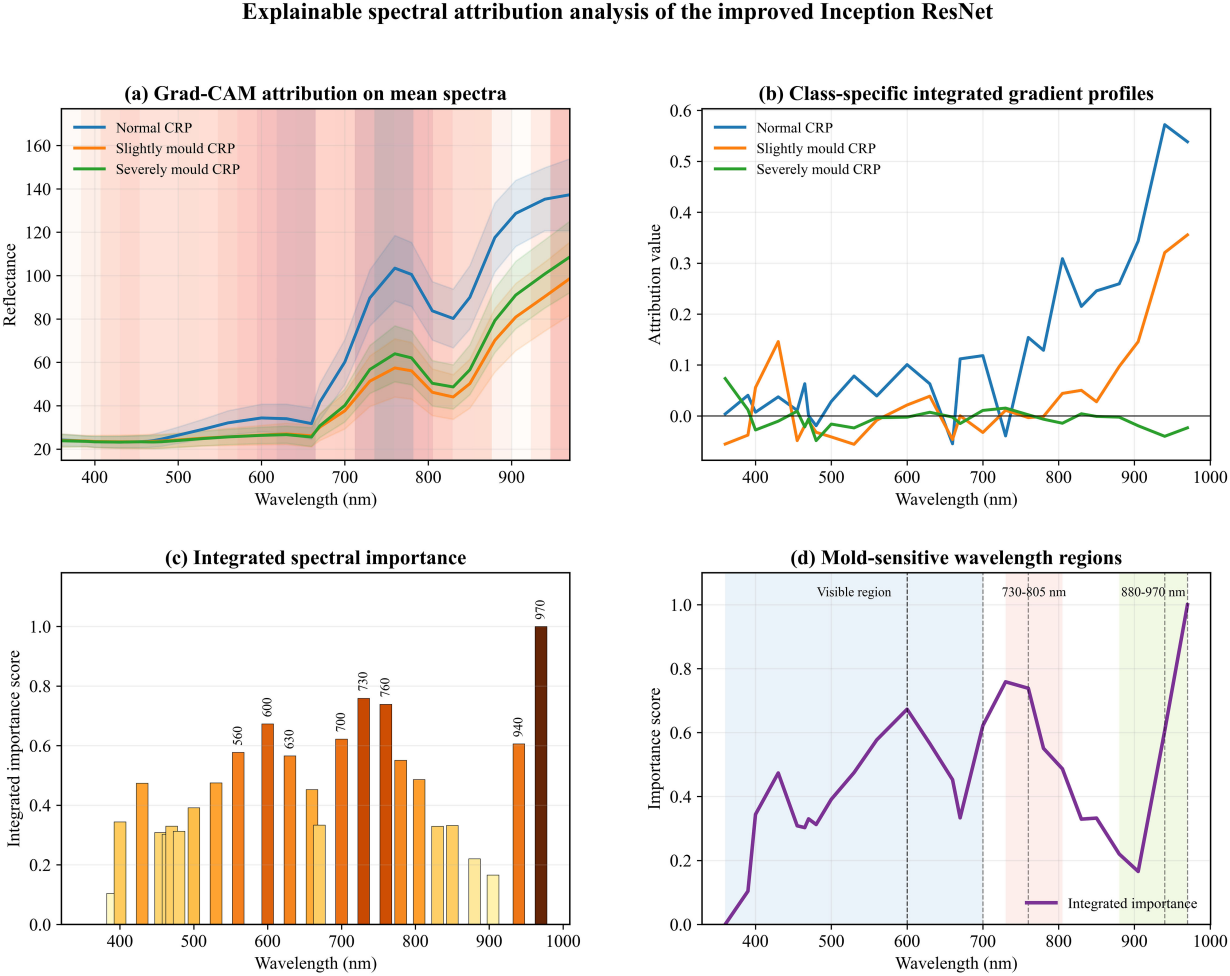
Explainable spectral attribution analysis of the proposed improved Inception ResNet. **(a)** GradCAM attribution map superimposed on the mean spectra. **(b)** Class-specific integrated gradient profiles of normal, slightly mould-deteriorated, and severely mould-deteriorated CRP. **(c)** Integrated spectral importance derived from Grad-CAM, integrated gradients, and attention-guided response. **(d)** Main mould-sensitive wavelength regions and their possible spectral assignments.

To reveal the decision mechanism of the proposed improved Inception ResNet, explainable spectral attribution analysis was conducted using 1D Grad-CAM, integrated gradients, attention-weight visualisation, and integrated spectral importance. The results are shown in **Figure 9**, and the main wavelength importance scores are summarised in **Table 9**. The attribution analysis was performed at the 26 available LED centre wavelengths; therefore, all reported important wavelengths correspond to actual centre wavelengths used by the multispectral system.

**Table 9:** Literature-supported interpretation of important centre wavelengths identified by integrated attribution analysis.

Rank	Wavelength (nm)	Integrated importance score	Spectral interpretation	Supporting literature
1	970	1.000	O–H/water-related absorption and high-humidity deterioration response	(Grassi et al., 2021; Zhang et al., 2024)
2	730	0.760	Tissue structure and scattering variation during deterioration	(Grassi et al., 2021)
3	770	0.740	Moisture redistribution and water-associated response	(Grassi et al., 2021; Zhang et al., 2024)
4	620	0.670	Visible colour and pigment-related surface reflectance change	(Grassi et al., 2021)
5	700	0.620	Visible-to-NIR transition response related to surface colour and scattering variation	(Grassi et al., 2021)
6	940	0.600	O–H/water-related overtone absorption associated with moisture-state change	(Grassi et al., 2021; Zhang et al., 2024)
7	570	0.580	Pigment and surface colour variation	(Grassi et al., 2021)
8	660	0.560	Surface colour response related to visible appearance change	(Grassi et al., 2021)
9	750	0.550	Moisture-related response and tissue/scattering change	(Grassi et al., 2021; Zhang et al., 2024)
10	805	0.490	Vis/NIR deterioration-related discriminative response	(Pang et al., 2025)

As shown in **Figure 9a**, Grad-CAM highlighted several wavelength regions that contributed strongly to the classification decision. The strongest attribution was mainly located around the visible colour-sensitive centre wavelengths, the 730, 750, 770, and 805 nm centre wavelengths, and the 940 and 970 nm centre wavelengths. This indicates that the proposed model did not depend on the whole spectral sequence uniformly. Instead, it focussed on several mould-sensitive centre wavelengths. These regions were consistent with the spectral differences observed in the mean spectral curves.

The class-specific profiles obtained from integrated gradients are shown in **Figure 9b**. Normal CRP showed a strong positive attribution in the near-infrared region, especially after 805 nm. Slightly mould-deteriorated CRP also presented positive attribution in the long-wavelength region, but its response was weaker than that of normal CRP. In contrast, severely mould-deteriorated CRP showed relatively low and stable attribution values. This result suggests that the model mainly used the decrease and redistribution of near-infrared spectral responses to distinguish different deterioration states.

The integrated spectral importance results in **Figure 9c** further confirmed the dominant contribution of several key centre wavelengths. The 970 nm centre wavelength showed the highest normalised importance score of 1.000. The centre wavelengths at 730, 770, 620, 700, and 940 nm also showed high importance scores, with values of 0.760, 0.740, 0.670, 0.620, and 0.600, respectively. These wavelengths were mainly located in the visible-to-near-infrared transition region and the long near-infrared region.

The mechanistic interpretation in **Figure 9d** showed that the important wavelengths could be divided into three main spectral regions. The visible-region centre wavelengths (570, 620, 660, and 700 nm) may be associated with surface colour change, pigment-related reflectance, and fungal coverage (Grassi et al., 2021). The 730, 750, 770, and 805 nm centre wavelengths may reflect moisture redistribution and tissue-structure/scattering variation during mould development (Grassi et al., 2021; Zhang et al., 2024). The 940 and 970 nm centre wavelengths are close to NIR regions associated with O–H and C–H related overtone/combination absorption responses and may reflect internal quality deterioration and water-related spectral changes (Grassi et al., 2021; Zhang et al., 2024). Previous CRP-specific NIR work and other mould-related spectral imaging studies also support that fungal development can induce discriminative spectral features captured by machine-learning or deep-learning models (Tan et al., 2024; Chun et al., 2024; Pang et al., 2025).

The explainability results demonstrate that the improved Inception ResNet learned meaningful spectral features rather than relying on random or non-specific responses. The highly weighted wavelengths were consistent with the known spectral changes caused by mould deterioration. Therefore, the proposed model shows both strong classification performance and reasonable spectral interpretability, supporting its potential use for rapid CRP storage quality monitoring.

### 3.8 Discussion of model effectiveness and process monitoring potential

As summarised in **Table 10**, previous studies have established spectral imaging as a nondestructive tool for food quality and safety assessment (Gowen et al., 2007; Feng and Sun, 2012; Qin et al., 2013), and CRP-specific NIR spectroscopy has confirmed that mould damage can induce detectable spectral changes (Tan et al., 2024). Compared with these studies, the present work extends CRP mould deterioration detection from single-point spectroscopy to Vis/NIR multispectral imaging and deep feature learning. The results are also consistent with spectral imaging studies of fungal infection in strawberry and mouldy peanuts, where deep-learning models captured subtle mould-related spectral responses and outperformed conventional machine-learning methods (Chun et al., 2024; Pang et al., 2025). At the model level, the advantage of the improved Inception ResNet is supported by previous evidence that deep models can learn nonlinear and hierarchical representations from hyperspectral data (Signoroni et al., 2019; Ahmed et al., 2024). Therefore, the combination of multi-scale convolution, residual learning, and attention-based refinement is suitable for capturing distributed mould-sensitive responses in CRP. Meanwhile, the remaining confusion between slightly and severely deteriorated samples suggests that CRP mould deterioration is a continuous process and should be further validated using broader storage conditions, more production batches, and quantitative microbial or chemical indicators.

**Table 10:** Literature-based comparison supporting the Discussion of CRP mould deterioration detection.

Reference	Object and method	Main relevance	Relation to this study
(Gowen et al., 2007; Feng and Sun, 2012; Qin et al., 2013)	Reviews of hyperspectral/multispectral imaging for food quality and safety	Spectral imaging can provide non-destructive spatial-spectral information	Provides the methodological basis for applying Vis/NIR imaging to CRP storage quality inspection
(Tan et al., 2024)	Portable NIR spectroscopy, PLS-DA, and selected wavelengths for mould-damaged CRP	CRP mould damage can induce detectable NIR spectral changes	The present study extends CRP detection from single-point spectroscopy to Vis/NIR multispectral imaging and deep feature learning
(Chun et al., 2024)	Hyperspectral fluorescence imaging and 1D CNN for early *Botrytis cinerea* infection in strawberry	Fungal infection can generate subtle spectral changes detectable by deep learning	Supports spectral detection of fungal deterioration, while CRP differs as a dried and heterogeneous product
(Pang et al., 2025)	Hyperspectral imaging and CNN-based models for aflatoxin quantification in mouldy peanuts	CNN-based models can outperform conventional machine-learning methods in mould-related spectral analysis	Supports the observed advantage of the improved Inception ResNet over conventional and baseline deep-learning models
(Signoroni et al., 2019; Ahmed et al., 2024)	Deep-learning review and agro-product hyperspectral reconstruction study	Deep models can learn nonlinear and hierarchical spectral representations when model design is appropriate	Explains why multi-scale convolution, residual learning, and attention refinement help capture distributed mould-sensitive responses

### 3.9 Limitations

Several limitations should be acknowledged. First, although the external validation samples were purchased from different commercial distributors two months later than the model-development samples, they still originated from the same representative production area; therefore, broader validation across different production regions, seasons, suppliers, storage environments, and imaging instruments is still needed. Second, the distinction between slightly and severely mould-deteriorated samples may involve transitional states, which explains the remaining spectral overlap between these two classes. Third, the current labels were mainly based on controlled storage treatment and expert assessment; quantitative microbial counts, mycotoxin analysis, or chemical compositional verification were not systematically included. Finally, the model was developed under laboratory imaging conditions, and future work should evaluate robustness under practical storage, multi-instrument transfer, and online inspection scenarios.

## 4 Conclusion

This study developed a Vis/NIR multispectral imaging method combined with an improved Inception ResNet for rapid and non-destructive detection of mould deterioration in CRP. A self-developed LED-based multispectral imaging system with 26 LED centre wavelengths was used for CRP spectral-image acquisition. Spectral analysis showed that mould deterioration caused clear reflectance changes, especially in visible colour-sensitive wavelengths and near-infrared water/tissue-related wavelengths. The t-SNE visualisation further confirmed that normal, slightly mould-deteriorated, and severely mould-deteriorated CRP samples showed distinguishable spectral distributions, although overlap remained between the two mould-deterioration stages. Among the preprocessing methods, SG provided the most effective spectral representation. The proposed improved Inception ResNet achieved the best classification performance compared with SVM, XGBoost, MLP, 1D CNN, ResNet, and the original Inception ResNet. The ablation results verified the contribution of multi-scale feature extraction and attention-based refinement. Independent-batch external validation further demonstrated the preliminary generalisation ability of the proposed method under the current source and storage-condition settings. Overall, Vis/NIR multispectral imaging coupled with the improved Inception ResNet provides a feasible tool for CRP storage quality monitoring, batch screening, and process quality control. Future work will focus on expanding sample sources, incorporating quantitative microbial and chemical indicators, and validating the method under practical storage, multi-instrument transfer, and online inspection conditions.

## Data Availability

The original contributions presented in the study are included in the article/supplementary material. Further inquiries can be directed to the corresponding author.
